# Cerebral small vessel disease: Recent advances and future directions

**DOI:** 10.1177/17474930221144911

**Published:** 2022-12-27

**Authors:** Hugh S Markus, Frank Erik de Leeuw

**Affiliations:** 1 Stroke Research Group, Department of Clinical Neurosciences, University of Cambridge, Cambridge, UK; 2 Department of Neurology, Radboud University Medical Center, Nijmegen, The Netherlands; 3 Center for Medical Neuroscience, Donders Institute for Brain, Cognition and Behaviour, Nijmegen, The Netherlands

**Keywords:** Cerebral small vessel disease, lacunar stroke, white matter hypersensitivities, vascular cognitive impairment

## Abstract

Cerebral small vessel disease (SVD) causes lacunar stroke and intracerebral hemorrhage, and is the most common pathology underlying vascular cognitive impairment. Increasingly, the importance of other clinical features of SVD is being recognized including motor impairment, (vascular) parkinsonism, impaired balance, falls, and behavioral symptoms, such as depression, apathy, and personality change. Epidemiological data show a high prevalence of the characteristic magnetic resonance imaging (MRI) features of white matter hyperintensities and lacunar infarcts in community studies, and recent data suggest that it is also a major health burden in low- and middle-income countries. In this review, we cover advances in diagnosis, imaging, clinical presentations, pathogenesis, and treatment.

The two most common pathologies underlying SVD are arteriolosclerosis caused by aging, hypertension, and other conventional vascular risk factors, and cerebral amyloid angiopathy (CAA) caused by vascular deposition of β-amyloid. We discuss the revised Boston criteria of CAA based on MRI features, which have been recently validated. Imaging is providing important insights into pathogenesis, including improved detection of tissue damage using diffusion tensor imaging (DTI) leading to its use to monitor progression and surrogate endpoints in clinical trials. Advanced MRI techniques can demonstrate functional or dynamic abnormalities of the blood vessels, while the high spatial resolution provided by ultrahigh field MRI at 7 T allows imaging of individual perforating arteries for the first time, and the measurement of flow velocity and pulsatility within these arteries. DTI and structural network analysis have highlighted the importance of network disruption in mediating the effect of different SVD pathologies in causing a number of symptoms, including cognitive impairment, apathy, and gait disturbance.

Despite the public health importance of SVD, there are few proven treatments. We review the evidence for primary prevention, and recent data showing how intensive blood pressure lowering reduces white matter hyperintensities (WMH) progression and delays the onset of cognitive impairment. There are few treatments for secondary prevention, but a number of trials are currently evaluating novel treatment approaches. Recent advances have implicated molecular processes related to endothelial dysfunction, nitric oxide synthesis, blood–brain barrier integrity, maintenance and repair of the extracellular matrix, and inflammation. Novel treatment approaches are being developed to a number of these targets. Finally, we highlight the importance of large International collaborative initiatives in SVD to address important research questions and cover a number which have recently been established.

Reducing the impact of cerebral small vessel disease (SVD) remains one of the major challenges in stroke medicine. Stroke itself is heterogeneous, with the most common ischemic pathologies being large artery atherosclerosis, cardioembolism, and SVD. Increasing evidence, including recent studies on genetic predisposition, demonstrates that these different subtypes of stroke have very different underlying pathophysiology and respond differently to treatments.^1^ While major strides have been made in preventing and treating both atherosclerotic and cardioembolic stroke, there has been little progress in treatment for SVD. Furthermore, the impact of SVD is greatly increased by its causal role in vascular cognitive impairment (VCI) and dementia.^2^ SVD not only causes lacunar stroke and intracerebral hemorrhage (ICH) but is also the most common pathology underlying VCI.^2^ It is also a contributor to the majority of cases of clinical dementia occurring in the elderly, in whom mixed pathology is the norm.^3^ In this issue of international journal of stroke (IJS), we focus on SVD, highlighting exciting new research in the area and also important remaining questions.

## What is SVD?

**Figure 1.**
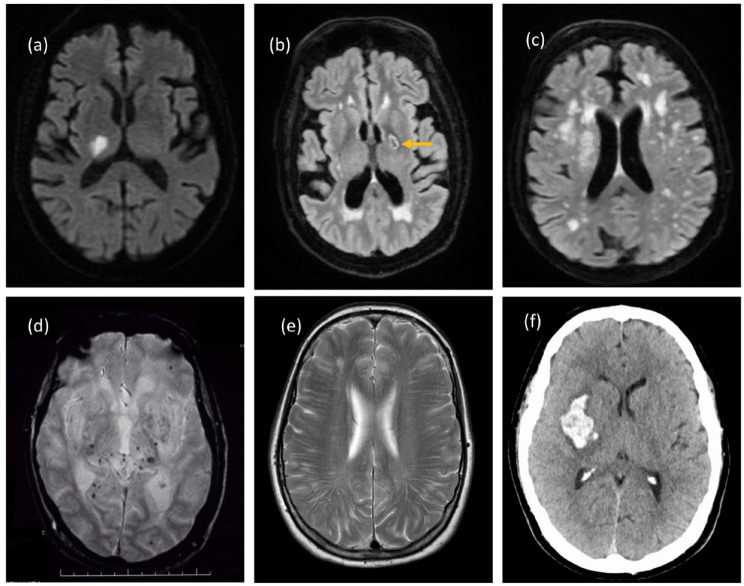
Imaging appearances of age-related non-amyloid SVD. (a) Acute lacunar infarct in right thalamus on DWI. (b) Old cavitated lacune in posterior limb of left internal capsule on FLAIR. (c) Confluent white matter hyperintensities on FLAIR. (d) Deep CMB on gradient echo scan. (e) Enlarged PVS intracerebral. (f) Basal ganglia hemorrhage on CT. Copyright Hugh Markus.

SVD refers to any pathologic process that damages small end arteries, arterioles, venules, and brain capillaries. Characteristic magnetic resonance imaging (MRI) features are used to define SVD, including lacunar infarcts, white matter hyperintensities (WMH), cerebral microbleeds (CMB), enlarged perivascular spaces, and brain atrophy (see Figure 1). Its most common clinical presentations are with lacunar stroke and cognitive impairment, but increasingly the importance of other features is being recognized. These include motor impairment, (vascular) parkinsonism, impaired balance, falls, and behavioral symptoms, such as depression, apathy, and personality change. Consequently, SVD is a major risk factor for transition to disability and a nursing home.

## The global burden of SVD

Epidemiological data show wide geographical variations in stroke incidence with a particular burden in low- and middle-income countries (LMICs),^4^ but there is much less data looking at individual stroke subtypes, including lacunar stroke. Talking to colleagues from many LMICs, our impression is that the burden of SVD in these countries is high, but there has been little published data to support this view. This makes the review in the current issue of IJS on the global burden of SVD by Lam et al.^5^ particularly timely. Reviewing the relatively limited data available, they find that the prevalence of radiological evidence of SVD is high in LMICs. They report a high prevalence of imaging features of SVD with moderate-to-severe WMH was 20.5%, 40.5%, and 58.4% in the community, stroke, and dementia groups, respectively. The median prevalence of lacunes was 0.8% and 33.5% in the community and stroke groups. This matches well with a previous systemic review based largely on data from high-income countries, which showed the presence of WMH ranged 65–96% and of lacunes, 8–31%.^6^ These data demonstrate the ubiquitous nature of SVD, particularly as we all age. Even in the absence of obvious clinical features, these radiological features of SVD are important, being strong predictors of both stroke and dementia risk.^7^ However, Lam et al.’s^5^ paper also highlights the paucity of information on the epidemiology of lacunar stroke and radiological SVD in LMICs; we need more studies both to determine its global prevalence and geographical variations, and prospective studies to determine whether this is changing over time.

## Etiology—the heterogeneity of SVD

Cerebral SVD is not a single disease but can be caused by diverse pathological processes. The two most common pathologies underlying SVD are arteriolosclerosis caused by aging, hypertension, and other conventional vascular risk factors, and cerebral amyloid angiopathy (CAA) caused by vascular deposition of β-amyloid. Other rarer causes include monogenic conditions, such as cerebral autosomal dominant arteriopathy with subcortical ischemic strokes and leukoencephalopathy (CADASIL), venous collagenosis, and postradiation angiopathy.

CAA is an age-related SVD, affecting cortical and leptomeningeal vessels, and is characterized pathologically by progressive deposition of amyloid-β in the cerebrovascular wall. CAA is the primary cause of lobar ICH and an independent contributor to age-associated cognitive impairment.^8–10^

Age-related non-amyloid SVD may also be heterogeneous. While diffuse arteriosclerosis affecting the small vessels is believed to play an important role, lacunar infarcts can also be caused by microatheroma at or near the origin of the perforating arteries. This was first suggested in seminal neuropathological studies by C. Miller Fisher in the 1960s,^11^ and is supported by different risk factor profiles,^12^ and more recently by direct visualization of the pathology in vivo using high-resolution 7T MRI.^13,14^

## Advances in diagnosis of CAA

**Figure 2.**
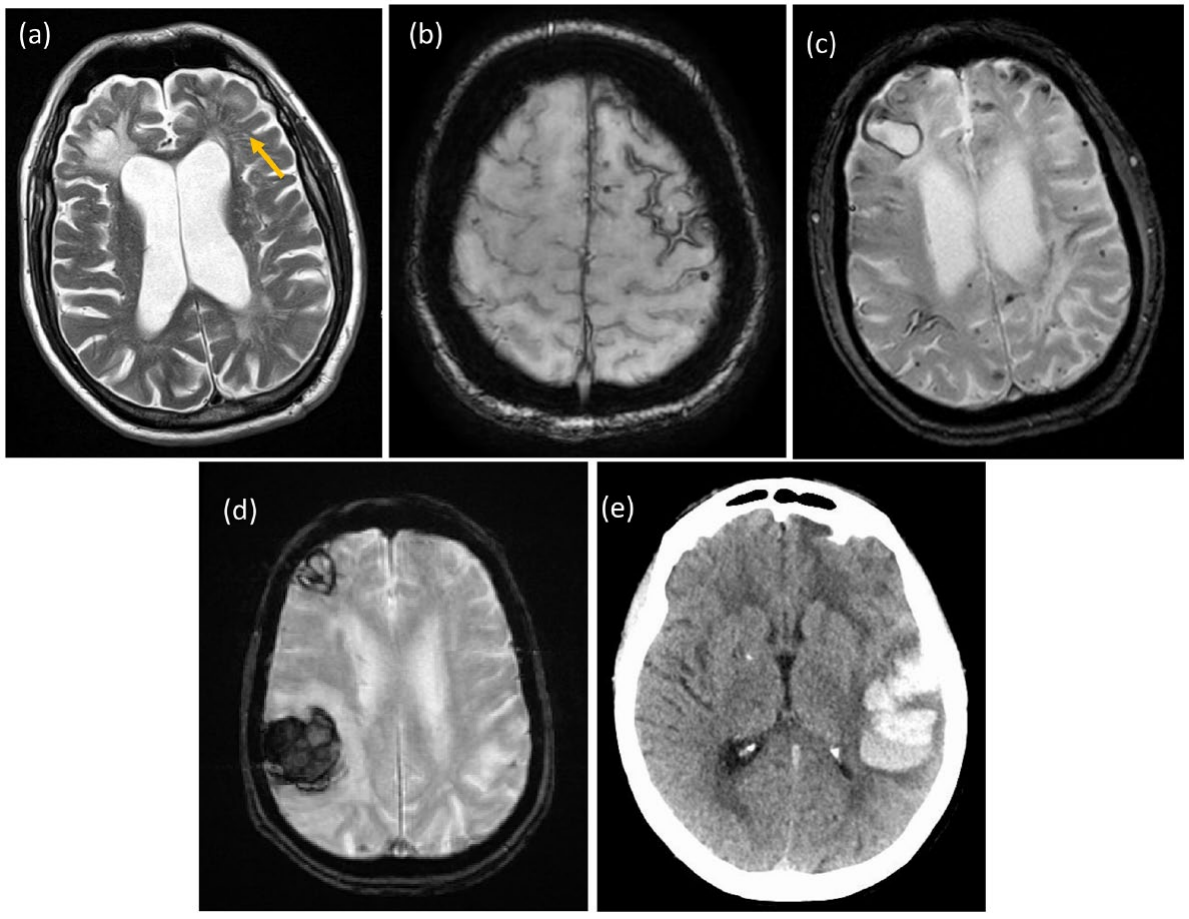
Imaging appearances of CAA. (a) Enlarged PVS (arrowed) and old right frontal ICH on T2 scan. (b) Superficial siderosis in right frontal region on susceptibility-weighted scan. (c) Cortical microbleeds in CAA on susceptibility-weighted scan. (d) Multiple cortical ICHs. (e) Lobar hemorrhage on CT. Copyright Hugh Markus.

An accurate diagnosis of CAA during life is important for both clinical care and enrollment of participants in research. The reference standard for CAA diagnosis remains histopathological analysis from brain autopsy or biopsy samples, but using MRI and incorporating markers seen on gradient echo or susceptibility-weighted imaging including CMB and superficial siderosis (Figure 2) non-invasive diagnostic criteria have been developed. A revised version of these Boston criteria has recently been validated and shown to have a sensitivity of 74.5% (65.4–82.4%) and specificity of 95.0% (83.1–99.4%) in patients who had autopsy as the diagnostic standard.^15^

MRI is not always available, and computed tomography (CT) biomarkers of CAA have been suggested and include both the presence of sulcal subarachnoid blood usually at the convexity, and of a lobar ICH with finger-like projections (FLPs). In this issue, Schwarz et al.^10^ studied 140 survivors of spontaneous lobar ICH with both acute CT and MRI. A high probability of CAA on the CT criteria showed a specificity of 87.2% (95% confidence interval (CI): 78.3–93.4) and a sensitivity of 29.6% (95% CI: 18.0–43.6) for probable CAA (vs non-probable CAA), defined by the modified Boston criteria; the area under the receiver operating characteristic curve (AUROC) was 0.62 (95% CI: 0.54–0.71). They conclude that in a hospital population, CT biomarkers might help rule-in probable CAA, but their absence is probably not as useful to rule it out, and that MRI is the optimal imaging modality in ICH survivors with suspected CAA.

## Insights from brain imaging

Imaging, particularly MRI, is transforming our understanding of SVD, improving both diagnosis and understanding of pathogenesis, and increasingly being used as a surrogate endpoint in treatment trials. In this issue, van den Brink et al.^16^ review these advances. They cover how advanced structural imaging techniques, including diffusion MRI, enable improved detection of tissue damage, including characterization of white matter appearing normal on conventional MRI. These techniques enable progression to be monitored and may be useful as surrogate endpoints in clinical trials.^17^ In the second section, they describe how advanced MRI techniques can demonstrate functional or dynamic abnormalities of the blood vessels, which could be targeted in mechanistic research and early-stage intervention trials. Such techniques include the use of dynamic contrast-enhanced MRI to measure blood–brain barrier (BBB) permeability, and MRI methods to assess cerebrovascular reactivity. In the third section, they describe how the increased spatial resolution provided by ultrahigh field MRI at 7T allows imaging of individual perforating arteries for the first time, allowing direct visualization of the pathology in vivo. Such studies have demonstrated perforating artery occlusion^18^ and altered branching patterns.^19^ More recently, 7T MRI has been adapted to measure flow velocity and pulsatility within the perforating arteries, opening up the possibility of studying how drug interventions improve vessel function.^20^

Recent imaging has also highlighted how SVD is more dynamic than previously appreciated. Serial diffusion-weighted imaging (DWI) scans have shown small, apparently asymptomatic DWI-positive lesions are much common than symptomatic lacunar infarcts.^21^ Their clinical significance, and whether they relate to disease progression, remains to be fully determined although a recent report associated them with greater radiological progression of SVD and cognitive decline compared with patients without DWI + lesions.^22^ It has also been suggested that WMH lesion volume may regress in some patients, and not only progress.^23^

## SVD and cognitive impairment

**Figure 3.**
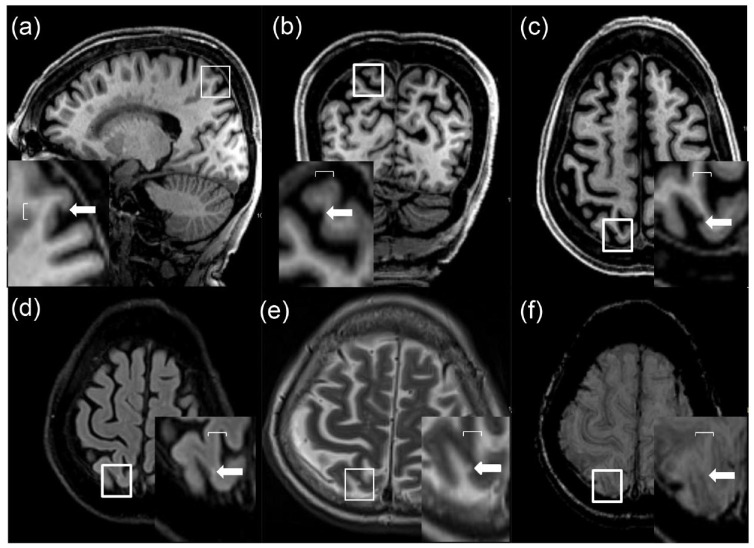
Cortical cerebral microinfarcts on 3T MRI. They are defined as hypointense cortical lesion found on T1-weighted images ((a) sagittal, (b) coronal, and (c) axial views) explored further as (d) hyperintense or isointense on FLAIR and (e) T2-weighted images. This was further confirmed by the absence of hypointense signal on (f) susceptibility-weighted image due to hemorrhagic lesion or vessel. Kindly provided by Saima Hila Copyright Saima Hilal.

Recent studies have provided important insights as to how SVD causes cognitive impairment. Lacunes, WMH, more diffuse white matter ultrastructural damage identified on DTI, and CMB have all been associated with cognitive impairment and dementia in SVD.^24–28^ In non-amyloid SVD, complex white matter networks, derived using DTI and tractography, have been shown to be disrupted. In cross-sectional studies, network disruption was found to mediate the effects of WMH, lacunes, and more diffuse white matter damage on cognition,^29^ and in prospective studies to predict future dementia risk.^30,31^ Atrophy has also been associated with cognition in non-amyloid SVD, particularly gray matter atrophy. It has been suggested that this occurs due to degeneration secondary to white matter track disruption;^32^ but more recently, it has been reported that the presence of cortical microinfarcts in SVD may also be involved. Initially visualized on high-field strength 7T MRI, these have now been shown to be detectable on 3T MRI as shown in Figure 3^33^ and have been associated with cognitive impairment.^34^ Less data are available on CAA, but recent studies suggest that similar mechanisms may be responsible. A paper by Durrani in this month issue reports that altered white matter diffusivity, cerebrovascular reactivity, and atrophy, taken together, accounted for about half the effect of CAA on cognition.^9^

While we have considerable data on risk factors for SVD itself, there are limited data on what are the risk factors for cognitive impairment in patients with SVD. A paper by Ohlmeier et al.^35^ also in this issue in almost 1000 lacunar stroke patients reports that diabetes mellitus (odds ratio (OR) = 1.98, 95% CI = (1.40–2.80), *p* < 0.001) and higher body mass index (BMI) (OR = 1.03, 95% CI = (1.00–1.05), *p* = 0.029) were independently associated with increased risk of VCI and years of full-time education with lower risk (OR = 0.92, 95% CI = (0.86–0.99), *p* = 0.018). An association between cognitive performance and diabetes has been identified previously,^36^ suggesting that diabetic control may be important in preventing VCI.

While SVD is the most common pathology underlying VCI, many other stroke pathologies, including cortical infarcts and ICH, can cause the condition. Progress on treating VCI has been disappointing. In response to this, also in this issue, Biesbroek and Biessels^37^ challenge our current thinking about the VCI. They argue that VCI is best seen as an umbrella term, caused by multiple different pathologies, and these different mechanisms will have implications for treatment approaches, which are likely to differ according to the causative pathology. For example, in SVD, treatment should focus on the underlying pathological processes causing white matter degeneration and on reversing the consequences of complex network disruption. The authors argue for a paradigm shift in our way of approaching VCI from diagnosis through treatment.^37^ Understanding mechanisms underlying cognitive impairment in SVD may help develop better treatments, although currently these are lacking.

## Manifestations of SVD beyond stroke and dementia

SVD has also been associated with depression, apathy, emotional lability, gait disturbance and falls, and urinary incontinence. These can present in the absence of lacunar stroke, and awareness of SVD as the possible diagnosis is important.

Recent data have highlighted the frequency of apathy in SVD, shown how it dissociates from depressive symptoms, and is associated with the degree of white matter ultrastructural damage on DTI.^38^ It has been hypothesized that it results from the disruption of complex white matter circuits, in a similar fashion to the hypothesized mechanisms underlying cognitive impairment.^39^ It is perhaps best seen as another symptom of the underlying cognitive impairment and has been closely associated with the presence of executive dysfunction,^40^ a cardinal neuropsychological feature of SVD. Unlike depression in SVD, which can respond to antidepressant therapy, treating apathy can be frustrating. A recent secondary analysis of a large trial assessing fluoxetine therapy following stroke found that fluoxetine was associated with a reduction in depressive symptoms but had no effect on apathetic symptoms.^41^ Cognitive approaches can alleviate the burden of apathy, but such symptoms remain disabling, often more so for the carer than the patient.^39,42^

Gait disturbances and SVD frequently coexist in the elderly, and increasing evidence suggests that this is due to a causal relationship. As outlined in a systematic review in this issue by Blumen et al.,^43^ a variety of gait impairments, including reduced speed, poor performance when carrying out a competing task, and gait apraxia, have been associated with SVD. A variety of MRI markers of SVD, including lacunar infarcts, WMH, CMB, brain atrophy, and reduced white matter integrity, are all associated with impaired gait.^43^ Recent studies suggest gait impairment also results from disruption of complex brain networks dependent on white matter pathways^44^ and showed that decline in global network efficiency over time was associated with gait decline.^45^ Importantly, Blumen et al.^43^ also show that abnormal gait in SVD is a predictor of subsequent cognitive decline and dementia.

## Enlarged perivascular spaces; a new player in SVD pathogenesis

Perivascular spaces (PVS) are physiological spaces surrounding small blood vessels as they run from the subarachnoid space through the brain parenchyma.(Figures 1(e) and 2(a)). Dilatation of PVS can be seen on MRI. They have a signal consistent with the cerebrospinal fluid and may be a marker of PVS dysfunction.^46^ PVS are believed to be important conduits for the removal of metabolic waste and maintenance of homeostatic fluid circulation in the brain as part of the “glymphatic system.”^47^ This has led to the suggestion that enlarged PVS reflect impairment of brain fluid and waste clearance. Increasing evidence suggests that they are another facet of SVD. They are frequently seen in patients with SVD and are associated with other markers of SVD including lacunes, WMH, CMB, and cortical microinfarcts.^48,49^ However, their independent contribution to the etiology of SVD needs to be established, as does their role in cognitive decline.^48^ It has been hypothesized that failure of brain fluid transport, via the glymphatic system, plays a key role in initiation and progression of SVD, and that stagnation of glymphatic transport may drive loss of brain fluid homeostasis leading to transient white matter edema, perivascular dilation, and ultimately demyelination.^50^ MRI techniques have been recently developed to examine lymphatic system dysfunction, using diffusion tensor image analysis along the PVS (ALPS index), and have shown altered glymphatic function in SVD.^51^

## Current treatment options

Despite the public health importance of SVD, and many patient and public consultation efforts identifying the consequences of SVD as a key priority for stroke research,^52^ there are few proven treatments for the disease.^53^

Hypertension is the most important risk factor for non-amyloid SVD, and recent primary prevention data suggest that rigorous control of hypertension may delay the onset of VCI and dementia. The most convincing data come from the SPRINT-MIND trial showing that intensive blood pressure lowering to a systolic of 120 mmHg was associated with both reduced WHM progression,^54^ and a reduction in the combined endpoint of mild cognitive impairment and dementia.^55^ This study shows the power of adding cognitive tests into larger cardiovascular prevention trials. We now need similar data to demonstrate whether control of other risk factors also reduces VCI risk.

In terms of secondary prevention following lacunar stroke, current strategies have been mostly inferred from studies of ischemic stroke in general, the majority of which did not specifically examine efficacy in lacunar stroke. Only one large definitive Phase-3 trial has focused exclusively on lacunar stroke patients, with MRI confirmation. The SPS3 trial (Secondary Prevention of Small Subcortical Strokes) showed in 3020 patients that long-term dual antiplatelet therapy with clopidogrel and aspirin was not superior to aspirin alone^56^ and that more intensive blood pressure lowering (target systolic < 130 mmHg) reduced ICH, and there was a trend toward reduced recurrent strokes.^57^ There has been concern about blood pressure reduction in patients with severe SVD, in whom cerebral autoregulation may be impaired. However, the recent PRESERVE trial, in patients with lacunar stroke and confluent WMH, showed no reduction in cerebral blood flow^58^ or any increase in white matter damage quantified on DTI, in patients randomized to a systolic blood pressure of 125 mmHg, compared with 140 mmHg.^59^ No trials similar to SPS3 have been performed to assess the effects of statins, smoking cessation, diabetes mellitus management, and lifestyle interventions on lacunar stroke.^53^

Unfortunately, little reliable information on preventing lacunes is available from other clinical trials because unlike SPS3, they generally have not required MRI for stroke subtyping. As many as half of the patients with a lacunar syndrome do not have SVD on more rigorous subtyping.^60^ However, trials specifically targeting lacunar stroke, or MRI features of SVD, are increasing (see Table 1). One interesting agent is cilostazol, a phosphodiesterase 3 inhibitor that inhibits platelet aggregation and improves endothelial dysfunction. It is already approved in major markets for other indications and has been the subject of several large trials in ischemic stroke (not limited to SVD) primarily in Asia, where in some it appeared to have lower bleeding risk than other antiplatelets. The Phase-2 LACI2 trial examined its efficacy in SVD in 400 lacunar stroke patients, in a two-by-two factorial design with isosorbide mononitrate.^61^ Results recently presented in December 2022 at the UK Stroke Forum showed no treatment effects but demonstrated feasibility for a larger Phase-3 trial.

**Table 1.** Recently completed, and ongoing, trials of emerging and novel therapies in SVD.

Category	Drug type	Study population	Outcome measure	Details	Results/status	Trial name and registration or reference
Antiplatelets	Cilostazol	Clinical lacunar stroke	Feasibility of Phase-3 trial	*N* = 400 Open, control, and active	Results presented in December 2022. No difference in outcome but phase 3 study shown to be feasible	LACI2 NCT03451591
Remote ischemic preconditioning	Remote ischemic preconditioning	Acute (within 7 days) lacunar infarct	Dynamic cerebral autoregulation	*N* = 100 Control and active	Ongoing	ESCAPE-SVD NCT05225948
	Remote ischemic preconditioning	Lacunar stroke on TOAST criteria	Flow-mediated dilation	*N* = 30 Crossover	Ongoing	NCT03635177
	Remote ischemic preconditioning	Lacunes and/or WMHs and/or CMBs on MRI	Change in WMH volume	*N* = 60 Control and active	Significant reduction in Fazekas and Schelten’s scores at 180-day (both p < 0.05) and 300-day (both p < 0.01) follow-ups in treatment arm only	RIC-SVD NCT04816500^84^
Phosphodiesterase-5 inhibition	Tadalafil	MRI evidence of lacunar infarct(s) and/or confluent WMH (⩾ grade 2 on Fazekas scale)	Flow velocity in large cerebral arteries, cortical brain oxygenation, endothelial function, and endothelial biomarkers	*N* = 20 Crossover Single dose	Increased blood oxygen saturation in cortex but no change in TCD CBV or endothelial function	ELTAS NCT02801032^85^
	Sildenafil	Minor stroke or TIA and WMH on MRI or CT	TCD-MCA velocity and reactivity. BOLD cerebrovascular reactivity	*N* = 75 Crossover	Ongoing	OxHARP NCT03855332
	Tadalafil	Lacunar stroke or TIA with MRI lacunes or WMH	CBF on ASL MRI	*N* = 55 Single-dose, double-blind cross-over	No treatment effect on CBF	PASTIS NCT0245025386
BBB permeability and neuroinflammation	Minocycline	Lacunar stroke and WMH	BBB permeability and microglial activation on PET–MRI	*N* = 44 Double-blind placebo and active	In analysis phase	MINERVA ISRCTN15483452
GLP-1 agonist	Exenatide	Age-related white matter changes scale of 2 or 3	DTI white matter integrity (PSMD)	*N* = 110 Control and active	Ongoing	GAPP-SVD NCT05356104
Neural regeneration	Mouse nerve growth factor (mNGF)	TIA or stroke with lacunar infract on MRI	Alzheimer’s disease assessment scale-cognition (ADAS-cog) score	*N* = 50 Randomized single blind	Recruiting	NCT04041349
Allopurinol	Allopurinol	Ischemic stroke	WMH progression rate over 2 years	*N* = 464. Double-blind randomized placebo controlled	Recruitment completed	XILO-FIST NCT02122718
Multiple actions	DL-3-n-butylphthalide	Cognitive impairment AND MRI evidence of SVD; (confluent WMH OR multiple lacunes (> 2) OR strategic infarct)	Cognitive scales	*N* = 64 Double-blind randomized placebo controlled	Ongoing	NCT03906123
Herbal medicine	Cerebral care granule or Yangxue Qingnao granule	WMH with two or more risk factors OR lacunar infarct	MOCA	*N* = 114 Control and active	Ongoing	CABLE NCT05578521

SVD: small vessel disease; WMH: white matter hyperintensities; CMB: cerebral microbleeds; MRI: magnetic resonance imaging; CT: computed tomography; PET: positron emission tomography; DTI: diffusion tensor imaging; mNGF: mouse nerve growth factor; ADAS-cog: Alzheimer’s disease assessment scale cognition; ASL: arterial spin labeling; MOCA: Montreal Cognitive assessment; PSMD: Peak width of skeletonized mean diffusivity; BBB: blood brain barrier; CBF: Cerebral blood flow; BOLD: Blood Oxygenation Level Dependent imaging; TCD: Transcranial Doppler; MCA: middle cerebral artery; TOAST: trial of ORG 10172 in acute stroke treatment stroke classification.

CTN numbers refer to registration on clinicaltrials.gov and ISCTRN number refers to https://www.isrctn.com/.

## Future treatment directions

A major factor underlying the lack of treatments for SVD has been a limited understanding of the underlying pathophysiology. However, with recent insights, a number of new treatment approaches are being evaluated. A recent framework for SVD progression, supported by existing biomarker studies (predominantly neuroimaging-based), proposes that early endothelial dysfunction leads to the disruption of the BBB with leakage of fluid and toxic plasma proteins into the vascular media and surrounding tissues, with secondary effects on vascular reactivity, pericyte function, oligodendrocyte proliferation, and perivascular fluid drainage pathways.^24^ A better understanding of the molecular pathways underlying these processes may lead to new targets for drug therapy. Current evidence implicates molecular processes related to endothelial dysfunction, nitric oxide synthesis, BBB integrity, maintenance and repair of the extracellular matrix (e.g. matrix metalloproteinases and their inhibitors), oxidative stress, mechanical stress, thrombosis, and inflammation.^62^ Drug interventions for VCI due to SVD are also be evaluated. DL-3-n-Butylphthalide (NBP) has been evaluated in a number of studies, and a recent systematic review concluded there was an improvement in cognitive scores, including MOCA and MMSE, but that more high-quality trial data were needed.^63^ It is thought to act via a number of mechanisms, including inhibiting oxidative stress responses, neuronal apoptosis and autophagy, regulation of central cholinergic function, and neuroplasticity.^63^

Emerging evidence from genetic studies and other ‘omic studies in both sporadic SVD and monogenic SVD is highlighting a number of novel molecular markers.^1,64,65^ A key theme is the disruption of the extracellular matrix and matrisome, resulting in impaired vascular responses and increased BBB permeability.^66^ Inflammation is also increasingly implicated.^67^ Both systemic inflammation and central nervous system inflammation have been demonstrated in SVD,^68^ while lacunar stroke itself has been shown to result in immune reprogramming which is associated with more rapid white matter disease progression.^69^ Systemic inflammatory markers were found to predict SVD progression in longitudinal studies.^70^ A pathway linking hypoperfusion and hypoxia to BBB disruption and inflammation has been proposed based on the studies in a rodent model of white matter ischemia.^71^ In the same rodent model, minocycline administration was associated with a significant reduction in white matter damage and improved behavioral and survival outcomes.^71^ Minocycline is known to have anti-inflammatory properties within the brain, reducing the activation of microglia and may be effective in stabilizing the BBB. Both increased BBB permeability detected using dynamic contrast-enhanced MRI and microglial activation detected by Positron emission tomography (PET) with the radioligand 11C-PK11195 have been shown in patients with lacunar stroke and confluent WMH.^68^ A Phase-2 trial is examining the effectiveness of minocycline on these PET and MRI markers in patients with SVD.^72^ Better understanding of the immune perturbations in SVD, and how they relate to disease progression, may allow development of more targeted treatments.

## SVD and ICH treatment implications

SVD causes most spontaneous ICH and is also an important cause of ICH in the young as highlighted by Periole in this issue.^73^ Not only is CAA an important cause of lobar ICH, but risk-factor-related non-amyloid SVD is also a major cause of deep ICH. Furthermore, CMBs occur in both forms of SVD and are associated with an increased risk of ICH, while recurrent ischemic stroke is common in patients with ICH due to SVD, raising questions as to optimal antithrombotic therapy. These diagnostic dilemmas are examined in a comprehensive review in this issue by Best et al.^74^ They summarize the evidence linking neuroimaging markers of SVD to antithrombotic and thrombolytic-associated ICH, with an emphasis on CMB. A pooled analysis of 38 studies comprising 20,322 patients prescribed antithrombotics after ischemic stroke or transient ischemic attack (TIA) found rapidly increasing ICH risk with CMB burden,^75^ and showed that a CMB-based model (MICON-ICH) outperformed existing bleeding risk models in ICH prediction.^76^ However, CMBs were also associated with ischemic stroke, with absolute incidence exceeding that of ICH regardless of CMB burden and distribution.^74^ Thus, current observational evidence suggests that CMBs should not preclude standard antithrombotic therapy after ischemic stroke or TIA. Best et al. conclude that following ICH, recommencing antiplatelets is probably safe in most patients, while the inconclusive results of recent randomized controlled trials recommencing anticoagulant use make recruitment to ongoing trials (including those testing left atrial appendage occlusion) in this area a high priority.

## Consensus efforts to improve research in the field

As in other areas of stroke research, large international collaborative ventures are vital to take the field forward. Recent European Stroke Organization consensus criteria not only have usefully summarized treatment options in SVD but have also highlighted the lack of current treatments, and the need for large-scale randomized controlled trials.^77^ Accurately defining populations for future research studies and clinical trials are essential. The STRIVE criteria have been important in defining the radiological features of SVD^78^ and updated STRIVE-2 criteria are currently being produced. Similarly, the modified Boston criteria for CAA have been developed and evaluated in consensus initiatives.^15^ The FINESSE framework has recently laid out a framework for designing clinical trials in SVD, including suggestions for inclusion criteria, and both clinical and validated surrogate endpoints.^79^ Consortia are also evaluating the predictive value of imaging markers of SVD in predicting future stroke and dementia risk, across multiple populations. These include Meta VCI Map consortium,^80^ OPTIMAL,^81^ and STROKOG.^82^ The DISCOVERY study (Determinants of Incident Stroke Cognitive Outcomes and Vascular Effects on Recovery) is a prospective, multicentre, observational, nested-cohort study of 8000 nondemented ischemic and hemorrhagic stroke patients enrolled at the time of index stroke from centers throughout North America and followed for a minimum of 2 years, with serial cognitive evaluations and assessments of functional outcome.^83^ Subsets are undergoing research MRI and PET. The overall scientific objective of this study is to elucidate mechanisms of brain resilience and susceptibility to post-stroke dementia. DISCOVERY will include all stroke subtypes, but this will include many cases of lacunar stroke, and therefore, it will provide important information on the progression of SVD to dementia and factors that influence it. Such large-scale initiatives are essential if we take the field forward.

## Conclusion

SVD has an enormous global impact. While treating risk factors such as hypertension will reduce risk, there are few treatments for established disease. However, recent advances in the understanding of pathogenesis, and large collaborative ventures, will hopefully improve this situation. This issue of IJS highlights some of the exciting work in the field. The IJS is an excellent global platform to share recent insights into SVD.
